# Personal

**Published:** 1900-11

**Authors:** 


### PERSONAL.

Dr. Ernest Wende, of Buffalo, was elected president of the American Electro-therapeutic Association, at its recent meeting held at New York. The next annual meeting will be held at Buffalo.

---

Dr. A. A. Hubbell, of Buffalo, was elected vice-president of the New York State Medical Association, at its late annual meeting held at New York City.

---

Dr. Thomas Lothrop, of Buffalo, was recently elected president of the board of managers of the State Normal and Training School, vice David F. Day, deceased.

---

Dr. Augustus G. Pohlman, U. of B. Class, 1900, house physician at the Deaconess's Hospital, has been appointed an instructor in anatomy at Cornell University. Dr. Pohlman has gone to Ithaca and entered upon his new work.

---

Dr. William B. May, of Buffalo, has been appointed by Health Commissioner Wende an inspector of milk in the health department, vice Dr. John H. Grant, resigned.

---

Dr. L. S. McMurtry, of Louisville, attended upon invitation the 22d annual meeting of the Chicago Gynecological Society, on Friday,

October 19, 1900. At the dinner held at the rooms of the Athletic Association, he responded to the first toast on the list,—The Blue and the Gray.

---

Dr. John A. Miller, M.Sc., Ph. D., F.C.S., of Buffalo, analytical and consulting chemist, has removed his office and chemical laboratory to Nos. 44 and 45 Lewis Block, 13½ East Swan Street, Buffalo. Dr. Miller is chemist to the State of New York.

---

Dr. John W. Cameron, of Buffalo, announces the change of his residence and office to 305 West Utica Street. Office hours: 7-9 a.m. and 7-8 p.m.

---

Dr. Charles T. Crance, U. of B., 1900, formerly assistant surgeon Soldiers' and Sailors' Home, at Bath, has become a member of the surgical staff of the Fitch Hospital, at Buffalo,

---

Dr. Sidney A. Dunham, of Buffalo, who has been spending a few weeks in New York, studying nervous diseases at the Vanderbilt clinic, as announced in the Journal for September, has returned and resumed his professional practice.

---

Dr. P. W. Van Peyma, of Buffalo, recently returned from a three-month trip in Europe, where he attended the International Medical Congress, the Paris Exposition, and otherwise was entertained during a pleasant vacation.

---

Dr. John H. Grant, of Buffalo, was on October 15, 1900, appointed assistant commissioner of agriculture for the Ninth Division. Dr. Grant retains his residence in Buffalo.

---

Dr. E. Wood Ruggles, of Rochester, announces his removal to 294 Alexander Street in that city, where he will limit his practice to skin and genito-urinary diseases. Hours: 10-1; 2-4 and 7-8. Telephone 4741.

---

Dr. Oran S. Smith, a homeopathic physician of Union Springs, N.Y., has been appointed by the trustees, assistant surgeon at the Soldiers' Home, Bath, N.Y.

---

Dr. W. G. Spiller, of Philadelphia, professor of nervous diseases at the Polyclinic, read a paper at the Academy of Medicine before the section on medicine, Tuesday evening, October 9, 1900, entitled, The involvement of the nervous system in malaria. Previous to the meeting Dr. Spiller was entertained at dinner at the Fillmore, by Drs. Julius Ullmann and A. E. Woehnert. Among the other guests were: Drs. Charles Cary, Roswell Park, Eli H. Long, William C. Krauss, Chauncey P. Smith, James W. Putnam, Eugene A. Smith, Grover W. Wende, H. U. Williams, F. S. Crego, Irving M. Snow, A. A. Jones, DeLancey Rochester and William Warren Potter.

